# Traumatic penile amputation: a case report

**DOI:** 10.1186/s12894-017-0285-4

**Published:** 2017-10-10

**Authors:** Tushar Patial, Girish Sharma, Pamposh Raina

**Affiliations:** 1 Department of General Surgery, Indira Gandhi Medical College & Hospital, Shimla, Himachal Pradesh 171001 India; 2 Department of Urology, Indira Gandhi Medical College & Hospital, Shimla, Himachal Pradesh 171001 India; 3 Department of Urology, Indira Gandhi Medical College & Hospital, Shimla, Himachal Pradesh 171001 India

**Keywords:** Case report, Microsurgery, Penile injury, Replantation, Traumatic amputation

## Abstract

**Background:**

Traumatic amputation of the penis is a rare surgical emergency. Although repair techniques have been well described in literature, failure of replantation and its causes are poorly understood and reported. Herein, we report the case of a 9 year old boy who underwent replantation of his amputated penis with delayed failure of the surgery, along with a discussion of recent advances in the management of this condition.

**Case  Presentation:**

A 9-year-old boy was referred to our hospital for traumatic amputation of the penis. Papaverine aided microsurgical replantation of the severed part was performed, but by 48 h, the glans became discoloured and necrosis set in by 4 days. Unfortunately, by day 12 two thirds of the re-implanted penis was lost along with overlying skin.

**Conclusion:**

Replantation of an amputated penis in a pediatric patient is a daunting task even for experienced surgeons. The vasodilatory effect of papaverine for vascular anastomosis is well described, but the use of a paediatric cannula for identification and instillation of papaverine into penile vasculature, has not been described for the repair of penile amputation. Despite its apparent failure, we believe this technique may be valuable to surgeons who might encounter this rare event in their surgical practice, especially in resource limited settings like ours.

## Background

Traumatic amputation of the penis is a rare surgical emergency. A systematic review of 80 cases from 1996 to 2007 reported only 37.5% of cases undergoing a successful replantation [1]. The main etiologies for penile amputation are self-mutilation, accidents, circumcision, assault and animal attacks. Back in the 1970’s, an epidemic of penile amputations was reported from Thailand, where women amputated their husbands’ genitalia for infidelity. That case series of 18 patients remains the largest till date [2].

We report the case of a 9 year old boy who underwent unsuccessful microsurgical repair for traumatic penile amputation.

## Case report

**Fig. 1:**
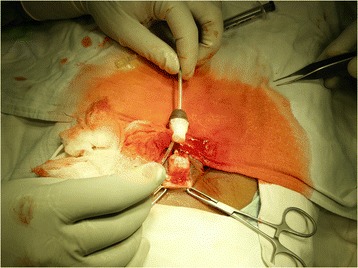
Photograph of amputated penis with stump remnant

A 9-year-old boy was referred to our hospital after his sister amputated his penis with a sickle. The sister was being treated for a mental illness, and the incident occurred after the two of them got into an argument while playing. After initial evaluation at a primary health center, the patient arrived at our hospital approximately 12 h after the injury. The amputated part of the penis was brought to us in a polythene bag immersed in muddied tap water. On examination of the genital area, the amputation had divided the penis into two halves. (Fig. 1).

**Fig. 2:**
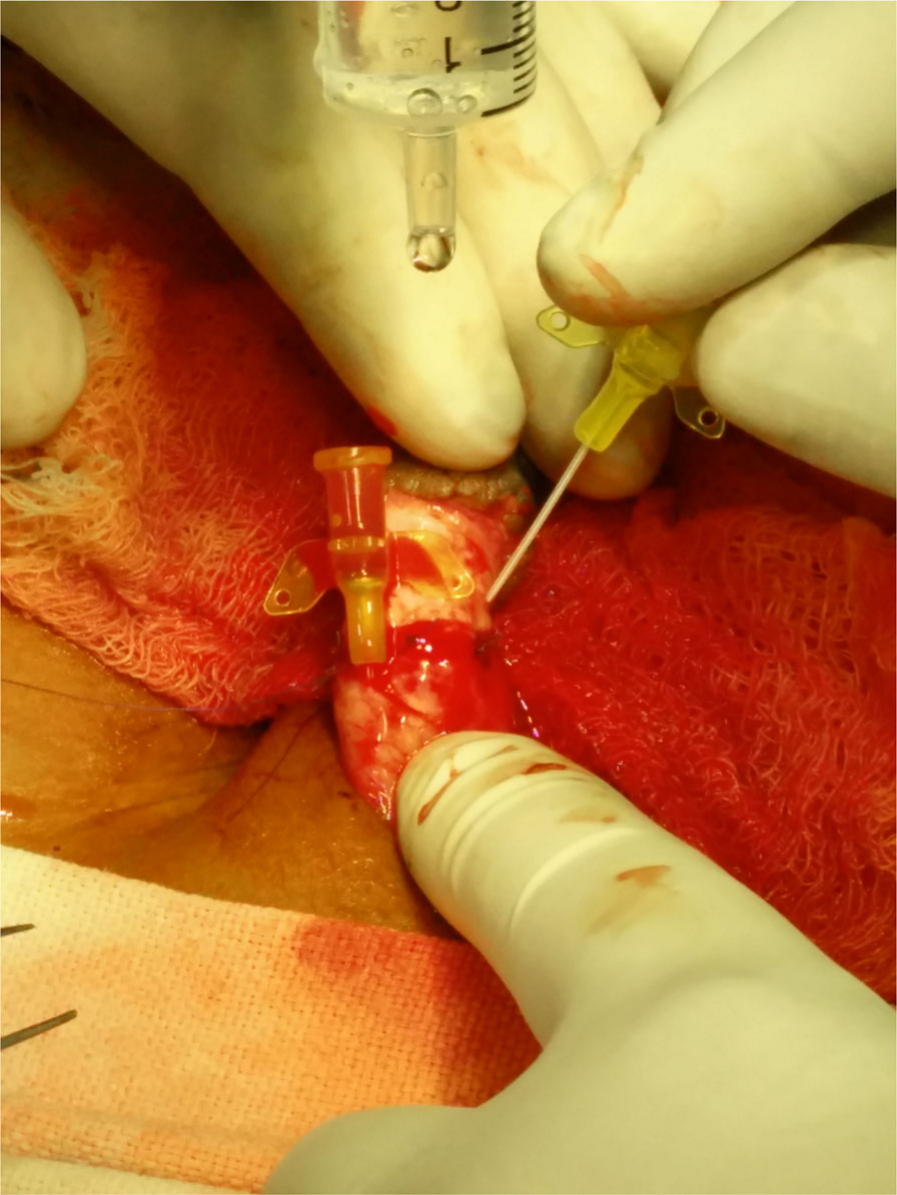
Cannulation of dorsal artery and delivery of papaverine

**Fig. 3:**
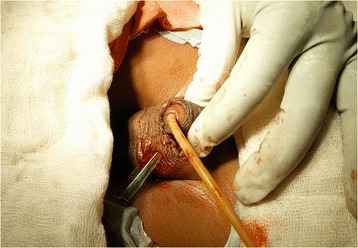
Replantation complete, with Foley’s catheter in situ and corrugated drain

After resuscitation, the patient was started on broad spectrum antibiotics and given tetanus toxoid for prophylaxis. Prior to replantation, the part was cleaned with normal saline and placed in an ice box for temporary storage. After carefully preparing the genital area, a 12 Fr Foley’s catheter was inserted through the amputated part and passed into the urinary bladder via the urethra at the amputated stump. The integrity of the corpus, urethra and dorsal vessels was verified. After orientation and alignment of the two ends an end-to-end anastomosis of the urethra was done using Vicryl 7–0. Next, the corporal bodies, were reattached using interrupted sutures of Vicryl 5–0, with special care taken near the dorsal aspect, to avoid injury to blood vessels. After carefully re-assessing the dorsal vein and artery, both vessels were cannulated with a 24 G cannula in succession and papaverine was injected to aid anastomosis. (Figure 2) Under loupe magnification, anastomosis of the dorsal vein and one dorsal artery was established using Prolene 9–0. Adequacy of the vascular anastomosis was confirmed by visible demonstration of the return of capillary refill at the glans and sustained bleeding from a deliberate needle puncture over the same area. Finally, the skin was closed with Prolene 3–0 sutures. (Figure 3) A corrugated drain was brought through just below the surgical site. No evidence of necrosis was seen during or immediately after the surgery. The wound was dressed with paraffin gauze and bacitracin ointment and the total duration of the surgery was just over 8 h. Post operatively, the patient was started on intravenous heparin. Monitoring was done with aPTT (activated partial thromboplastin time) and clinical examination performed every 8 h. By 48 h, the glans had become discoloured, with prolonged refill time as evidenced at the glans. The skin had also darkened, and necrosis set in by 4 days. No vascularity was found on Doppler examination. The patient was observed for 12 days. During this time, a Psychiatric consultation was done for both the victim and his sister. Despite best efforts, two thirds of the implanted penis was lost along with its overlying skin. The patients’ family refused further investigations and hence the necrotic tissue could not be sent for histopathological examination.

## Discussion

The first case of macroscopic penile replantation was reported in 1926 by Ehrich without repair of neurovascular structures. This fallacy was repudiated by the year 1977 due to the advancement of microsurgical techniques [1]. Regardless of technique, the two primary objectives for the treatment of any penile amputation are, preservation of penile length and maintenance of erectile as well as voiding functions [2].

Replantation of the penis is dependent on the condition of the stump and the amputated segment. If wound conditions at the amputation site are not favourable, debridement and closure of stump, followed by secondary reconstruction is preferable [3].

Successful replantation entails the following principles; removal of debris, debridement of necrotic tissue, anastomosis of the severed urethra, repair of corporal bodies and tunica, and microsurgical repair of the dorsal neurovascular plexus. When repaired without the use of a microscope there is a higher rate of erectile dysfunction and urethral strictures [4].

All patients presenting as cases of assault should be resuscitated and stabilized, before considering repair. A secondary survey should be mandatory to rule out other injuries.

In all cases an attempt to salvage the severed penis should be made [4]. The penis should be washed clean of all debris and placed in normal saline, and then placed in ice. Care should be taken to avoid the severed part being in direct contact with ice. Hypothermia increases the ischemia time and successful replantation has been reported as late as 16 h [2].

The opioid alkaloid Papaverine is well known for its vasodilatory effects and is commonly used during microsurgical repair. As a topical agent, it primarily acts as a phosphodiesterase inhibitor inhibiting the myosin light chain kinase [5]. A secondary mechanism of calcium antagonism has also been proposed for the agent [6]. The drug takes between 1 to 5 min to take effect and is known to reverse as well as prevent vasospasm [5].

Common complications of penile replantation include penile deformation, erectile dysfunction, hematoma formation, abscess, urethral fistula, urethral stenosis, delayed/ absent penile sensation and failure of replantation [4]. Since mental illness is often associated with these cases, a thorough psychiatric evaluation is a must for all patients, including those with failed implantation for possible future issues with body image and sexuality [2].

There are many causes for a failed anastomosis. These include, hematoma formation, intimal fibrosis, arterial spasm, destruction of the intima from trauma and bleeding in the media. The factors responsible for failed microvascular anastomosis can be classified as, surgical factors (e.g experience, fatigue, technique), the diameter of vessels and pre-existing vessel damage [7].

Due to the lack of histopathological evaluation of the necrosed part, any attempt at trying to identify the potential cause for anastomotic failure would be pure speculation. However, in our opinion, the presence of soil debris, the small diameter of vessels to be anastomosed, physical manipulation by the child and the time from injury till completion of repair, approximately 20 h, were major factors precluding successful anastomosis.

Post anastomosis monitoring, has been traditionally done by visual assessment. However, this was considered unreliable because of factors such as ambient temperature, vasospasm and positioning. To provide an objective evaluation, newer methods like transcutaneous oxygen measurement, Doppler flowmeters, implantable venous Doppler monitoring and transit-time flow monitoring have been proposed. Traditional intraoperative methods for anastomosis monitoring have been, clinical evaluation, optical visualization and in some centres Doppler examination. An upcoming real time method is the phase-resolved Doppler optical coherence tomography, which has already undergone animal trials at the Johns Hopkins University [8].

## Conclusion

Traumatic penile amputation is a rare emergency and despite being well described in literature, it is poorly understood. The use of papaverine as a vasodilator for vascular anastomosis is well described. But the use of a paediatric cannula for instillation of the drug into the penile vasculature, has not been described for repair of penile amputation. An operative microscope may not be available at many centres due to limited resources. Despite its limitations, a loupe based repair can provide acceptable results. We believe, this technique may be valuable to surgeons who might encounter this rare event in their surgical practice.

## Abbreviations

aPTT: Activated partial thromboplastin time
Fr: French
G: Gauge
